# Supplementary material for: Impact of Fermentation Pretreatment on Drying Behaviour and Antioxidant Attributes of Broccoli Waste Powdered Ingredients
Source: Foods. 2023 Sep 22;12(19):3526. doi: 10.3390/foods12193526 (PMC10572841; doi:10.3390/foods12193526)
Supplement: Supplementary file 1 [file foods-12-03526-s001.zip › foods-2624491-supplementary.pdf]

Supplementary Information

Fermentation pretreatment affects the drying response and antioxidant properties of broccoli wastes powdered ingredients

Claudia Bas-Bellver [1], Cristina Barrera [1*], Noelia Betoret [1] and Lucía Seguí [1]

[1] Institute of Food Engineering-FoodUPV (IIA-FoodUPV). Universitat Politècnica de València, Camino de Vera, s/n, 46022, Valencia, Spain; clbabel@etsiamn.upv.es (C.B.-B.); noebeval@tal.upv.es (N.B.); lusegil@upvnet.upv.es (L.S.)
* Correspondence: mcbarpu@tal.upv.es (C.B.); Tel.: +34 629 987 104

Results of the fermentation preliminary study

Figure 1 shows the results of the microbial counts of *Lactiplantibacillus plantarum* (LP) along the fermentation of ground (G) or chopped (C) broccoli stems. Microbial count increased during the first 24 h, when the maximum counting was obtained. Further measurements showed a decrease in microorganism population, probably due to nutrients depletion.

A decrease in pH values (from 6.3 to 4.1) after 96 h of fermentation was also registered, as a result of the formation of organic acids [27]. Considering that the recommended dose for probiotics to exert a beneficial health effect is 10^9 CFU/day [13,70], an ingesta between 1 and 5 g of 24 h-fermented broccoli stems would provide the expected benefit (Figure S1).

Multifactor analysis of variance confirmed that the intensity of tissue disruption did not significantly influence microbial growth. Thus, ground broccoli fermented during 24 h was chosen for further drying studies, according to this and previous results [18].

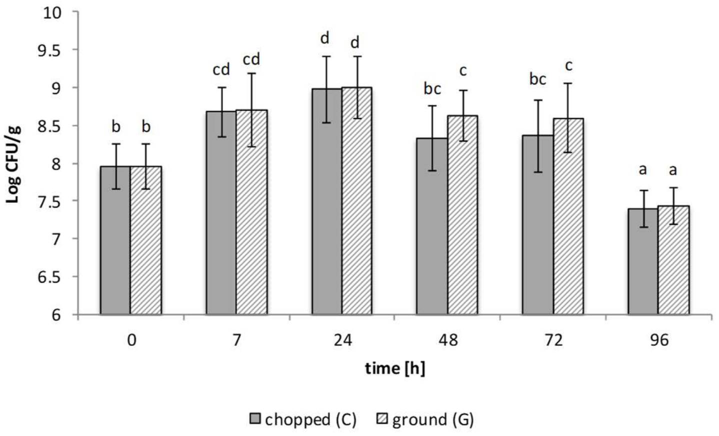

Figure S1. Viable counts of *L. plantarum* during fermentation of chopped (C) and ground (G) broccoli stems. Error bars represent the standard deviation of four replicates from two replicas. [a,b,c…] Different letters in the same series indicate statistically significant differences at the 95% confidence level (p-value < 0.05).
